# Intestinal Epithelium and Autophagy: Partners in Gut Homeostasis

**DOI:** 10.3389/fimmu.2013.00301

**Published:** 2013-09-30

**Authors:** Sarron Randall-Demllo, Marcello Chieppa, Rajaraman Eri

**Affiliations:** ^1^Mucosal Biology Laboratory, School of Human Life Sciences, University of Tasmania, Launceston, TAS, Australia; ^2^Laboratory of Experimental Immunopathology, IRCCS “de Bellis”, Castellana Grotte (BA), Italy

**Keywords:** IBD, autophagy, intestinal epithelium, ATG16L1, IRGM

## Abstract

One of the most significant challenges of cell biology is to understand how each type of cell copes with its specific workload without suffering damage. Among the most intriguing questions concerns intestinal epithelial cells in mammals; these cells act as a barrier between the internally protected region and the external environment that is exposed constantly to food and microbes. A major process involved in the processing of microbes is autophagy. In the intestine, through multiple, complex signaling pathways, autophagy including macroautophagy and xenophagy is pivotal in mounting appropriate intestinal immune responses and anti-microbial protection. Dysfunctional autophagy mechanism leads to chronic intestinal inflammation, such as inflammatory bowel disease (IBD). Studies involving a number of *in vitro* and *in vivo* mouse models in addition to human clinical studies have revealed a detailed role for autophagy in the generation of chronic intestinal inflammation. A number of genome-wide association studies identified roles for numerous autophagy genes in IBD, especially in Crohn’s disease. In this review, we will explore in detail the latest research linking autophagy to intestinal homeostasis and how alterations in autophagy pathways lead to intestinal inflammation.

## Introduction

Autophagy literally means “self-eating.” Autophagy describes a process by which our cells are able to cope with damaged organelles and malformed proteins by directing them to lysosomal degradation. Autophagy may be divided into three classes; macroautophagy, macroautophagy, and chaperone-mediated autophagy (1, 2). Of these three classes, macroautophagy, or simply autophagy, as it will be referred to hereafter, is the most studied and is the form implicated in the regulation of chronic inflammation and is therefore the focus of this review. Induction of autophagy promotes cell survival during nutrient deprivation, oxidative stress, and endoplasmic reticulum (ER) stress, and is therefore critically important for the maintenance of cellular homeostasis (3–5). Since autophagy involves the degradation of bulk cytoplasm it is minimally active under normal conditions but may be readily induced by a diverse range of intracellular stressors. It is now evident that the mechanisms which target specific structures for degradation are in fact quite selective. Selective degradation of mitochondria (mitophagy), ribosomes (ribophagy), peroxisomes (pexophagy), and ER has been reported (6–9). An additional form of selective autophagy is xenophagy. Xenophagy serves to degrade invasive pathogens in a specialized form of autophagy termed xenophagy (10). Xenophagy involves the recruitment of the autophagic machinery to degrade invasive viral or bacterial pathogens. The myriad roles of autophagy in regulating homeostasis make it of particular interest in study of the intestinal mucosa, where all the aforementioned stresses are likely to converge.

The intestinal mucosa is constantly exposed to dietary and microbial antigens. The intestinal epithelium and its associated mucus layer serves as a physical barrier against the external environment. However, in order to fulfill its function in the absorption of nutrients a degree of permeability to the luminal contents is required. Naturally, not all antigens to which the intestinal mucosa is exposed are pathogenic; the correct balance between inflammation and tolerance is a complex mechanism that involves hematopoietic and non-hematopoietic cells. A form of intestinal homeostasis allowing the protection against pathogens whilst tolerating food antigen and the beneficial, non-pathogenic commensal microbes is required. The need to maintain intestinal homeostasis has seen a number of complex signaling pathways evolve between a diverse range of cell types. Deficiencies in this signaling network may result in a loss of homeostasis and in the development of chronic inflammatory diseases such as the inflammatory bowel diseases (IBD). Two chronic relapsing and remitting disorders, namely Crohn’s disease (CD) and ulcerative colitis (UC), constitute the majority of IBD cases. Both of these conditions cause severe abdominal discomfort and lead to significant morbidity and loss of quality of life. Currently no cure for IBD exists; the treatment of IBD involves the alleviation of symptoms using anti-inflammatory agents such as steroids and non-steroid anti-inflammatory drugs (NSAID). Despite intensive research, the exact pathogenesis of IBD remains elusive.

Recently, a number of studies, genome-wide association studies (GWAS) in particular, have identified a link between genes involved in autophagy regulation and IBDs. Given its importance of autophagy as a regulator of cellular homeostasis and of innate immunity, understanding the contribution of defective autophagy to the pathogenesis of chronic inflammation may be of considerable value to human health. In this review, we aim to initially discuss the general mechanisms of autophagy, and then describe in detail the link between autophagy machinery and the intestinal epithelium in gut homeostasis and inflammation.

## Autophagy Mechanisms

Although autophagy has been reported since the 1960s, detailed description of the molecular mechanisms regulating autophagy has only occurred relatively recently (11). We know now that the autophagy pathway is reliant upon the proteins encoded by autophagy-related genes (Atg), the first of which, Atg1, was identified 20 years ago in the yeast *Saccharomyces cerevisiae* (12). Since then, over 30 Atg proteins and their functions have been identified (13, 14). The autophagy-related genes essential for the assembly of the autophagosome are highly conserved between yeasts, worms, flies, and mammals. Such high degree of conservation is presumably due to the importance autophagy in cell survival, therefore much of our knowledge of autophagy mechanisms obtained from yeast may be translated to mammalian cells. Several comprehensive reports detailing the current understanding molecular mechanisms and regulation of autophagy in physiology and disease in both yeasts and mammals already exist in the literature (15, 16). For the purpose of this review however, we will give a brief overview of the proposed general mechanisms of mammalian autophagy prior to describing the role of autophagy-regulating genes in the pathogenesis of CD.

**Figure 1:**
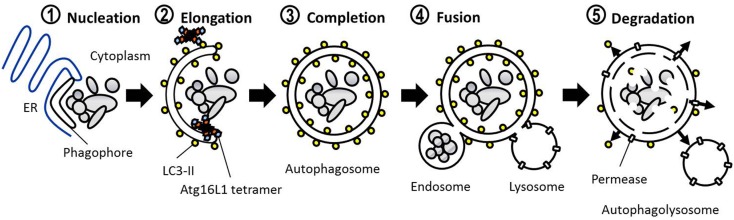
**Basic steps involved in mammalian macroautophagy**.

**Figure 2:**
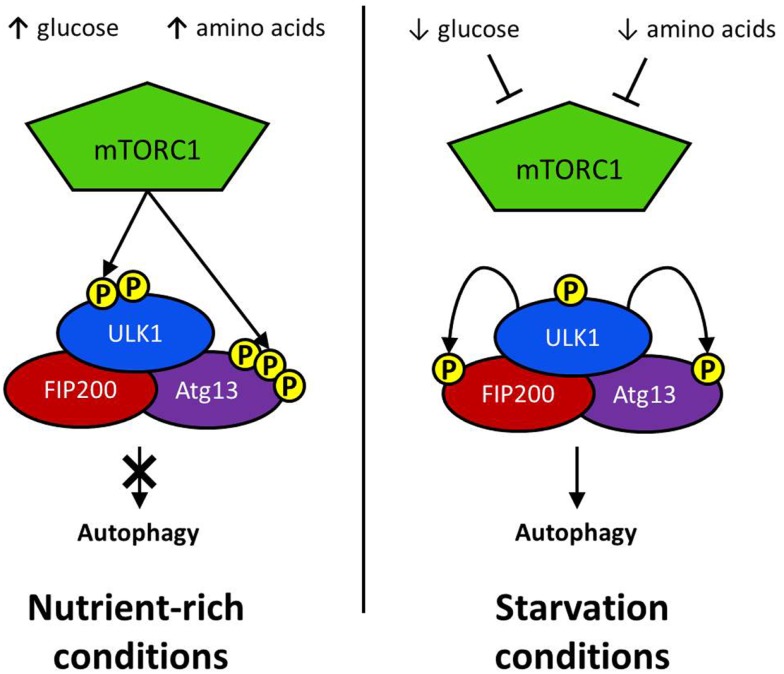
**Initiation via ULK1 complex: the regulatory complex mTORC1 represses autophagy activation in nutrient rich conditions**. mTORC1 phosphorylates a serine residue on ULK1 to prevent it interacting with positive regulators of autophagy induction. Atg13 activation is similarly repressed by mTORC1-mediated phosphorylation. Glucose or amino acid starvation results in the repression of mTOR activation. Consequently, ULK1 phosphorylates both FIP200 and Atg13, resulting in the activation of downstream autophagy effector proteins.

The defining feature of macroautophagy, as opposed to the other classes of autophagy, is the formation of the double-membrane vesicle known as the “autophagosome.” The process of autophagy may be divided into several stages: induction, nucleation, elongation, endosomal/lysosomal docking and fusion with the autophagosome, and finally, degradation (Figure 1). The first of these stages, the initiation of autophagy, may occur through a range of signaling pathways, dependent upon the stimulus. The mammalian target of rapamycin complex 1 (mTORC1) appears to be the central regulator of autophagy induction. In nutrient-rich conditions mTORC1 is active, and represses autophagosome formation (Figure 2). Inactivation of mTORC1, e.g., by starvation, results in the de-repression of signaling pathways downstream of mTORC1 and results in initiation of autophagy. The importance of mTOR in autophagy stimulated by other stressors such as certain invasive pathogens however, may be limited (17). Under the control of mTORC1 is a complex composed of uncoordinated 51-like kinase 1 (ULK1; the mammalian ortholog of Atg1), Atg13, Atg101, and focal adhesion kinase family interacting protein of 200 kDa (FIP200; Atg17 ortholog) (18–20). The ULK1-Atg13-FIP200 complex is thought to be the earliest factor recruited to the autophagosome precursor. Repression of mTORC1 results in phosphorylation of Atg13 and FIP200 by ULK1 and the entire complex is relocated to the phagophore (21, 22). Activation of Atg13 and FIP200 is required for the formation of the phagophore under starvation conditions whereas ULK1 appears to be dispensable (23). It remains to be seen whether the role of ULK1 in autophagy extends beyond its kinase function. The ULK1 ortholog in yeast, Atg1, interacts with the lipid membranes of vesicles via its C-terminal domain, suggesting that it may recruit the first vesicles to the phagophore assembly site (PAS) following autophagy induction (24).

The second step in the autophagic process involves the formation of a phospholipid bilayer membrane known as the isolation membrane or phagophore. This early membrane structure is the precursor to the mature autophagosome membrane. The origin of the autophagosome precursor, known as the “phagophore,” is an aspect of autophagy about which little is currently known. Considerable divergence in the formation of the phagophore between mammalian and yeast cells exists. In *S. cerevisiae* autophagosome formation begins at a defined location known as the PAS (25). The PAS is associated with the yeast vacuole and the resultant autophagosome eventually fuses with the vacuole and the autophagosomal contents are degraded. In contrast, mammalian autophagosomes may instead form at multiple locations throughout the cell (26).

Autophagosome formation has been observed associated with various membranous structures such as the ER, plasma membrane, Golgi apparatus, and mitochondria (27–30). A growing body of evidence supports the ER as a starting point for phagophore formation in mammalian cells. Axe et al. identified a unique compartment of the ER involved in autophagosome formation marked by the presence of phosphatidylinositol-(3)-phosphate (PI (3)P)-binding double FYVE-containing protein (DFCP1), since termed the omegasome (27). Visualization of the DFCP1^+^ omegasome shows the formation of the phagophore surrounded by the cradle-like omegasome. Furthermore, the two membranes are directly connected suggesting that perhaps the phagophore arises from the ER (27, 31, 32). Further experiments are required to determine the precise origins of the initial isolation membrane and the functional significance of the DFCP1^+^ omegasome in autophagy.

**Figure 3:**
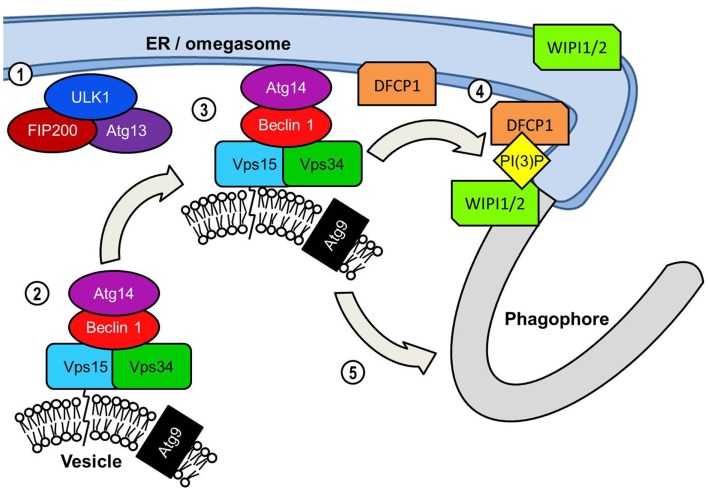
**PI(3)K-mediated vesicle nucleation: The activated ULK1 complex translocates to the phagophore formation site**. In starvation-induced autophagy this is likely to be adjacent to the ER initially but ULK1 also associates with the phagophore. ULK1 complex activation results in the translocation of the Beclin 1 lipid kinase complex to the phagophore assembly site. Beclin 1 acts as a scaffold protein forming a complex with Vps34 PI(3)K, its Vps15 subunit and Atg14. Similarly to orthologs yeast proteins the mammalian Vps15 subunit is predicted to interact with lipid membranes thereby tethering Vps34 to vesicles. Vps34 is the subunit of the Beclin1 lipid kinase complex that catalyzes the phosphorylation of the 3 region of the inositol head group of phosphatidylinositol to generate PI(3)P. The transmembrane Atg9 is found localized to post-Golgi vesicles but is depicted on the same vesicle as the Beclin 1 complex for convenience. The extent to which vesicular Beclin 1 and Atg9 overlap in their distribution is unclear. The inclusion of the Atg14 protein recruits the Beclin 1 complex specifically to the ER. This may serve two functions in assembly of the autophagosome. The first and perhaps most crucial function is the generation of PI(3)P at the omegasome and/or phagophore, which is essential for the recruitment of many effector proteins required for autophagosome assembly. PI(3)P production results in an accumulation of PI(3)P-binding protein DFCP1 at the omegasome membrane, the functional significance of which is unknown. ER-associated WIPI proteins, WIPI1 and WIPI2 also bind PI(3)P and are essential in the transition from omegasome to the double membrane phagophore.

An early event in the formation of the phagophore membrane is likely to be the recruitment of PI(3)P to the membrane. Although its precise role of PI(3)P in autophagy is unknown, it is a key requirement for the recruitment of many of the downstream effector proteins critical for extension and completion of the autophagosomal membrane (33). The autophagosome membrane is enriched with PI(3)P, particularly at the elongating tips of the phagophore (34, 35). Localization to these points most likely serves to recruit FYVE, PX, and WD-40 domain-containing autophagy effectors for efficient assembly of the phagophore membrane. Generation of PI(3)P requires the kinase activity of class III PI(3)K complexes to the phagophore (Figure 3). The most prominent of this class of PI(3)K complex in mammalian autophagy is the complex consisting of beclin 1 (BCN1), the PI(3)K Vps34, its regulatory subunit Vps15, and Atg14 (36). Following the localization of the ULK1 complex to the phagophore the next major step involves the recruitment of the beclin 1 complex to the phagophore. Atg14 is of critical importance in directing the beclin 1-Vps34 kinase to the phagophore due to its ability to interact with the ER membrane (35, 37, 38). Since the phagophore appears to form in close proximity to the ER membrane it is possible that the omegasome promotes autophagosome formation by recruiting PI(3)K activity to the adjacent membrane.

The kinase activity of Vps34 is also required for the activation of another key step in the process of autophagosome formation. WD-40 repeat domain-containing phosophoinositide interacting proteins (WIPI) WIPI1 and WIPI2 (mammalian orthologs of the yeast Atg18) may be found localized to the ER, plasma membrane, and Golgi (39). WIPI1 and WIPI2 have also been observed in close proximity to DFCP1 suggesting that it is recruited to the omegasome or phagophore via its PI(3)P-binding domain (40–42). Cells deficient in WIPI2 displayed defective DFCP1^+^ omegasome formation in response to starvation (42). WIPI1 and WIPI2 are both later found localized to the LC3^+^ autophagosomal membrane supporting the theory that WIPI1 and WIPI2 regulate the transition of the omegasome to phagophore (39). In yeasts the WIPI ortholog, Atg18, interacts with both Atg2 and Atg9, and is involved in retrograde movement of Atg9 from the autophagosome (43). Orthologs of the yeast Atg2, mammalian Atg2A and Atg2B are known to be essential for the completion of the autophagosome in mammalian autophagy but their role is still poorly understood (44). However, Atg9 has been the subject of considerable investigation due to its unique transmembrane localization and its role in the early stages of autophagosome formation. Interaction between Atg9 and the phagophore membrane appears to be transient yet essential for autophagy (45, 46). Atg9 is likely carried on Golgi-derived secretory vesicles or on endosomes, which are trafficked between the phagophore and the trans-Golgi network (TGN) and endosomes (29, 47). The trafficking of Atg9^+^ vesicles from other membranous organelles and the essential role of Atg9 in autophagosome formation has led to speculation that these vesicles may transport lipids vital for extending the phagophore (48). An additional role for Atg9 has been suggested by study into autophagy-induced by *Salmonella typhimurium*. Kageyama et al. observed that Atg9 is required for recruitment of the PI(3)K complex to the *Salmonella*-containing vacuole and formation of the autophagosome (49). The observed absence of PI(3)P-binding WIPI1 from membrane-bound bacteria suggested that WIPI1 may instead function downstream of Atg9.

**Figure 4:**
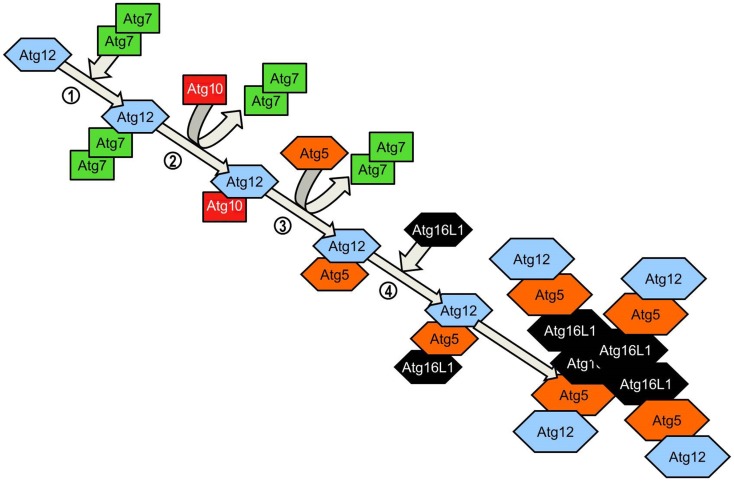
**Atg5-Atg12 complex conjugation system**. Agt12 is conjugated to Atg5 and Atg16L1 via a ubiquitinylation-like process. Homodimeric Atg7 functions as an ubiquitin-activating (E1)-like enzyme, activating Atg12 through the formation of a thioester bond between the Atg7 active site cysteine and the Atg12 C-terminal glycine. Atg7 transfers the activated Atg12 to the E2-like enzyme Atg10 via the C-terminal thioester linkage. Atg10 catalyzes the formation of an irreversible isopeptide bond between the Atg12 C-terminal glycine and Atg5. After the formation of the Atg5-Atg12 conjugate, Atg16L1 associates with the complex, binding non-covalently with Atg5. Interaction between Atg16 coiled-coil domains on adjacent Atg5-Atg12-Atg16L1 conjugates results in the formation of tetrameric complexes.

**Figure 5:**
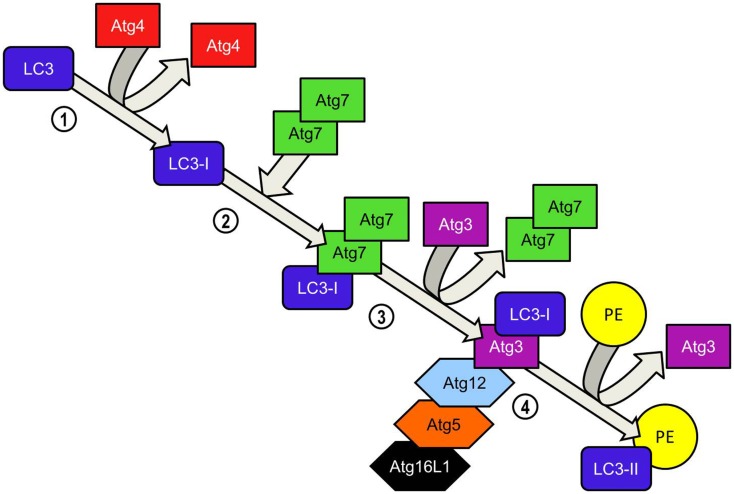
**LC3-PE conjugation system**. Atg4 cleaves the carboxyl terminal region of LC3 immediately after synthesis, generating a soluble LC3-I which now possesses a C-terminal glycine required for further modifications. Homodimeric Atg7 functions similarly to ubiquitin-activating (E1) enzymes and binds the LC3-I C-terminal exposed by Atg4 before recruiting Atg3 via its N-terminal domain. LC3-I is transferred to the Atg3 enzyme during the Atg3-Atg7 interaction. Atg7 dissociates from the LC3-I complex. LC3-II is formed when LC3-I is conjugated to the lipid phosphatidylethanolamine (PE) in a reaction catalyzed by Atg3. Binding of the Atg5-Atg12-Atg16L1 to Atg3 enhances the lipidation of LC3-I. The ability of the Atg5-Atg-12 complex to bind PE already inserted into the isolation membrane ensures that newly converted LC3-II is incorporated into the elongating isolation membrane. Unlike the formation of the Atg5-Atg12 complex, the conversion of LC3-I to LC3-II is reversible. Atg4 cleaves PE from LC3-II and LC3 is recycled.

Elongation of the phagophore/early autophagosome membrane in yeasts requires the protein Atg8. In mammals there have been four Atg8 orthologs identified; microtubule-associated protein 1 light chain 3 (LC3), GABARAP, GATE-16, and Atg8L. Of these four proteins LC3 is the best characterized in autophagy. LC3 is unique in that, once converted from LC3-I to its lipidated form (LC3-II) it is incorporated into the elongating autophagosome and remains associated with the autophagosome even after the dissociation of other Atg proteins. Thus LC3-II serves as an accepted and widely used marker of autophagosomes (50). For the efficient processing of LC3 from pro-LC3 to LC3-II, eukaryotes have evolved a pair of ubiquitinylation-like reactions. The first ubiquitinylation-like reaction catalyzes the formation of an Atg16L1-Atg5-Atg12 complex (Figure 4) (51). The Atg7 homodimer forms a thioester bond between a cysteine residue within the Atg7 active site and the Atg12 C-terminal glycine, similar in its mechanism to an ubiquitin E1-like activating enzyme. The Atg12 C-terminal thioester bond is subsequently transferred from Atg7 to Atg10 (E2-like enzyme). Atg10 catalyzes the formation of an isopeptide bond which covalently links Atg5 to Atg12. Atg16L1 binds non-covalently to Atg5 and enables the formation of a Atg16L1-Atg5-Atg12 tetrameric complex through interaction between Atg16L1 coiled-coil domains (52). The second ubiquitinylation-like reaction involves the conversion of lipidated LC3-II from its precursor form (Figure 5). The protease Atg4 cleaves the C-terminal region of pro-LC3 immediately after the precursor is synthesized, releasing a soluble LC3-I into the cytosol (53). The proteolytic action of Atg4 exposes a C-terminal glycine residue which allows for interaction with the active site of Atg7 homodimers. Atg7 functions as a E1-like enzyme in this second ubiquitinylation-like reaction also, this time however, transferring the LC3-I C-terminal domain to Atg3 (54). Atg3 (E2-like enzyme) catalyzes the conjugation of LC3-I to the lipid phosphatidylethanolamine (PE) (55). Lipidation of LC3 is a critical step in the formation and completion of the autophagosomal membrane (56). The Atg16L1 complex may facilitate the lipidation and incorporation of LC3 into the expanding autophagsomal membrane. The Atg16L1-Atg5-Atg12 conjugate interacts with Atg3 and may also lipidate LC3 independent of Atg3 (57). The Atg16L1 complex may therefore assist in ensuring LC3 conversion occurs in close proximity to the membrane to accelerate autophagosome formation.

The final stage in autophagy involves the fusion of endosomes and lysosomes with the mature autophagosome resulting in degradation and recycling of its contents. Dissociation of Atg proteins from the autophagosomal membrane may be required before fusion with endosomes or lysosomes can begin. Part of this process involves the depletion of PI(3)P associated with the autophagosome. Defective PI(3)P phosphatase activity prevents the dissociation of Atg proteins from the autophagosome and results in an accumulation of completed autophagosomes in the yeast cytoplasm (58). In mammals the fusion of endosomes with the autophagosome forms a multivesicular body (MVB), which appears to be an important process in the degradation of select protein accumulations (59). Fusion of endosomes is mediated by various proteins involved in endosomal trafficking including ESCRT, SNARE, and Rab7 proteins (59–61). The protein encoded by the ultraviolet radiation resistance-associated gene (UVRAG) regulates Rab7-mediated endosomal trafficking by forming a unique complex with beclin 1-Vps34-Vps15 (62). The association of UVRAG with beclin 1 recruits the Vps kinase complex to the membrane, thereby activating Rab7 promoting endosomal and lysosomal fusion with the autophagosome. Recent evidence suggests that LC3-II bound to the autophagosome membrane may also be a key element in Rab7-mediated endosomal/lysosomal fusion (63). Following lysosomal fusion, the inner autophagosomal membrane and its contents are degraded and amino acids transported into the cytoplasm. In contrast to yeasts which utilize Atg proteins such as Atg15 and Atg22 in the degradation and recycling process, to our knowledge no such equivalents have yet been discovered in mammalian autophagy. Instead common lysosomal proteases such as cathepsins B, D, and L are required for degradation of autophagosomal contents (64, 65). Recycling of nutrients from the degraded autophagosomal contents are presumably recycled via lysosomal permeases transferred to the autophagosomal membrane during lysosomal fusion. However, a recent study has identified a mammalian transmembrane lysosomal permease, Spinster, involved in transporting carbohydrate from the autophagosome into the cytoplasm (66). Of particular interest is the role of Spinster in the termination of autophagy, which may be marked by autophagic lysosome reformation (ALR), during which functional lysosomes are reconstituted from the autophagosome (67). Spinster is necessary for the reactivation of mTOR and the subsequent reformation of functional lysosomes from the autophagolysosome (66). The role of other lysosomal efflux transporters in signaling the termination of autophagy and its potential significance in human disease may be a promising area for future study.

## Autophagy Genes Involved in Intestinal Homeostasis and Susceptibility to IBD

To date a number of polymorphisms in a wide range of genes have been identified as risk factors predisposing an individual to the development of IBD. Numerous genome-wide association scanning (GWAS) studies have attempted to identify the genes contributing to the development of CD. Two of the most prominent associations with CD have been autophagy-related gene 16-like (*ATG16L1*) and *NOD2* (68–71). The identification of an *ATG16L1* polymorphism as a genetic risk factor for CD by GWAS studies has drawn attention to the potential importance of autophagy as an etiologic factor in IBD. Since the identification of *ATG16L1* as a risk factor for CD, more recent GWAS studies have implicated a number of other autophagy-related genes in susceptibility to CD (summarized in Table 1). Of particular interest in the etiology of CD are the genes which link innate immunity with autophagy such as *IRGM* and *NOD2*. It is noteworthy that autophagy appears to play a lesser role in UC. ER stress has been implicated in the development of UC and as such a strong association with XBP1 mutants and UC exists. However, one of the mechanisms which may be employed to relieve ER stress is autophagy, and as such defective XBP1 affect autophagy induction in UC. However, limited overlap in susceptibility genes between IBD does exist, with the autophagy-regulating gene, *PTPN2* associated with both CD and UC (72).

**Table 1:** **Genetics of aberrant autophagy in IBD**.

Gene	Function	Polymorphism and association with IBD
*ATG16L1*	Required for assembly of the autophagosome	SNP encoding T300A substitution conferring susceptibility to CD (rs2241880) (67, 68, 72)
*NOD2*	Initiator of autophagy in response to MDP; recruits ATG16L1 to site of bacterial entry	Three SNPs associated with susceptibility to CD (rs2066844, rs2066845, and rs2066847) (69, 70, 115)
*XBP1*	Leucine-rich repeat transcription factor associated with autophagosome maturation	Several polymorphisms associated with CD (93, 116, 117)
*IRGM*	Small anti-microbial GTPase that regulates autophagosome formation	Two SNPs (1 exonic; rs10065172 and 1 deletion upstream of *IRGM*: rs13361189) associated with CD (67, 88, 90, 118)
*LRRK2*	Leucine-rich repeat kinase associated with autophagosome maturation	Association with CD (69, 97)
*ULK1*	Kinase that forms part of a key autophagy-initiation complex	SNP associated with CD (94)
*PTPN2*	Regulates formation of the autophagosome in response to IFN-γ and TNF-α	Two SNPs (rs2542151 and rs1893217) with modest association with CD (67, 69, 70, 88), and UC (71)

### ATG16L1

Several GWAS studies have linked polymorphisms in the gene encoding the mammalian ortholog of Atg16 (*ATG16L1*) to susceptibility to CD (69, 73). Functional *ATG16L1* is crucial for the induction of autophagy. Atg5-Atg12 protein conjugates interact with Atg16L1 to form a high molecular weight protein complex essential for the elongation of the autophagosomal membrane (52, 74). Insight into the potential importance of *ATG16L1* and autophagy in the pathogenesis of CD were revealed by the generation of the following two experimental models.

#### Atg16L1 deficiency

Due to the importance of autophagy in neonatal survival, very few mice bred with a deficiency in Atg16L1 survived 1 day post-partum (75). To examine the effect of an otherwise lethal Atg16L1 deficiency, two experimental approaches have been used. The first involves the creation of chimeric mice, in which fetal liver cells from Atg16L1-deficient mice were transferred to lethally irradiated wild-type mice. In contrast to wild-type embryonic cells, fibroblasts deficient in functional Atg16L1 failed to respond to starvation with autophagosome formation (75). Furthermore, Atg16L1-deficient fetal liver chimeric mice displayed normal bacterial colonization of the colon and no indication of spontaneous colitis (75). However, Atg16L1-deficient chimeric mice administered 5% DSS for 7 days exhibited a marked exacerbation of colitis with reduced survival.

#### ATG16L1 hypomorphic mice

The second model utilized gene trap mutations of the *ATG16L1* gene resulting in hypomorphic gene expression. In addition to defective autophagosome assembly, ileal Paneth cells from *ATG16L1* hypomorphic mice displayed abnormal exocytosis and secretion of anti-microbial peptides (76). The morphological abnormalities in Paneth cells generated by this mutation were similar to that obtained by targeted deletion of epithelial *ATG5* (76). Apart from aberrant Paneth cell morphology, no histological differences could be observed between the ileum of *ATG16L1* knockouts and wild-type mice. The results of these experiments strongly suggest that Paneth cells in particular are vulnerable to defects in autophagy.

The validity of these murine models is supported by observations in human patients with CD. Defective Paneth cell function in Atg16L1-deficient mice resembles the abnormalities in Paneth cell secretory granule exocytosis in patients with CD (76, 77). The function of autophagy mechanisms including Atg16L1 may therefore to extend to cytokine secretion. Pro-inflammatory stimuli such as lipopolysaccharide and muramyl dipeptide (MDP) are capable of enhancing IL-1β transcription, thereby increasing levels of intracellular pro-IL-1β (78). The major mechanism through which IL-1β regulation occurs is at the post-translational level, cleavage of pro-IL-1β to the biologically active IL-1β. Cleavage occurs through the activity of the enzyme caspase-1, which itself must first be activated by the inflammasome protein complex. The greatly enhanced IL-1β secretion in Atg16L1-deficient macrophages suggests that autophagy plays a role in modulating immune responses from these cells. Indeed autophagy has been demonstrated to regulate IL-1β secretion *in vitro*.

Recently, Lee et al. (79) showed that increased MDP-induced IL-1β secretion from Atg16L1-deficient PBMC was greatly diminished by type III PI3K autophagy inhibitors. Another more recent study has revealed an additional autophagy-dependent regulatory mechanism in IL-1β signaling. The protein p62 is thought to promote the secretion of IL-1β via the activation of TRAF6, an element of the IL-1β receptor signaling pathway, upstream of NF-κB (79). Degradation of p62 via Atg16L1-mediated autophagy would therefore be expected to act as a negative regulator of IL-1β signal transduction. A reduction in functional Atg16L1, such as may be expected to occur with the CD-associated *ATG16L1* allele, may therefore lead to cellular hyper responsiveness to IL-1β secretion.

It seems plausible that autophagy mechanisms are required for secretion of Paneth cell granules via a non-conventional secretory pathway. Patients with CD carrying the *ATG16L1* T300A risk alleles also displayed increased numbers of LC3^+^ autophagosomes within Paneth cells (77). Increased numbers of autophagosomes would suggest that either formation of autophagosomes is increased or alternatively, lysosomal fusion is inhibited and as a result autophagosomes accumulate in the cytoplasm. Defective ATG16L1 may therefore inhibit the completion of the autophagosome membrane by unknown mechanisms. However, Thachil and colleagues reported that neither the common *ATG16L1* nor *IRGM* polymorphisms associated with CD, appeared to be responsible for the increased numbers of autophagosomes in Paneth cells (77). It is possible that other ATG16L1 variants exist, in the coiled-coil domain region for example, which would prevent efficient autophagosome degradation (80). Further characterization of the CD-associated ATG16L1 mutant proteins will likely explain the conflicting data. Overall, evidence still suggests that ATG16L1 mutation results in a consequent reduction in lysozyme secretion. Therefore defective autophagy could conceivably reduce the anti-microbial effectiveness of the intestinal barrier, permitting greater microbial invasion into the mucosa.

Exposure to the microbiota or an inappropriate response to the microbiota of the intestine is widely considered to be a causative factor in IBD. In DSS-induced colitis for example, inflammation may be improved by antibiotic therapy (81). Other experimental models of colitis such as T cell transfer and IL-10^−/−^ colitis do not display overt inflammation when housed in sterile conditions. *ATG16L1* hypomorphic mice, when rederived into a sterile barrier facility, displayed morphologically normal Paneth cells (82). The evidence for a microbial etiological factor was strengthened further by inoculating mice with a murine norovirus (MNV). The CR6 strain causes persistent gastrointestinal infections in mice. Inoculation of *ATG16L1* hypomorphic mice with the CR6 strain reproduced the Paneth cell abnormalities observed in mice housed under less stringent conditions. Inoculation of a similar strain (CW3) that did not result in persistent infection, also failed to induce alteration to Paneth cell morphology. Interestingly when infected hypomorphic mice were challenged with a moderately high dose of DSS the resultant pathology was strikingly similar to CD. These mice exhibited transmural inflammation of the anorectal junction accompanied by mucosal lymphoid aggregates, thickening of the muscularis propria, and subserosal fibrosis (82). In addition to colonic inflammation CR6-infected Atg16L1-deficient mice also displayed ileal inflammation not observed with DSS challenge in wild-type mice. Inflammation of the ileum and colon could be attenuated by treatment with antibiotics, tumor necrosis factor-α (TNF-α), or interferon-γ (IFN-γ) blocking therapy, all of which can be used effectively in symptomatic treatment of CD. The experimental evidence suggests that chronic viral infection, when combined with epithelial injury and defective autophagy, may be the prelude to IBD.

The individual contribution of reduced functional Atg16L1 to inflammation in the MNV-DSS model remains to be elucidated. It is known that macroautophagic mechanisms are involved in immunity. It is possible that persistent enteric infections caused by some pathogens are poorly limited when macroautophagic mechanisms are deficient. However, alternative autophagy pathways may compensate in the absence of functional macroautophagy.

### NOD2

The nucleotide-binding oligomerization domain-containing (NOD) pattern recognition receptors NOD1 and NOD2 are key elements in immunity against intracellular pathogens. Cytoplasmic NOD1 and NOD2 recognize cell wall components of invasive bacteria such as *Shigella flexneri* and *Listeria monocytogenes*. The association of NOD2 variants with susceptibility to CD has highlighted the role of autophagy in innate immunity. The 3020insC SNP (rs2066847) frameshift mutation in *NOD2* is the most prevalent of the three identified NOD2 risk variants in patients with CD (83). This mutation produces a Nod2 protein with a truncated leucine-rich repeat (LRR) domain (84). The consequence of this mutation is a loss of Nod2 functionality. Expression of the 3020insC NOD2 variant in human cells resulted in attenuated activation of NF-κB in response to MDP or peptidoglycan (85). Not only is NF-κB activation abrogated but NOD2-mediated xenophagy is also affected.

Both NOD1 and NOD2 appear to be critical components in degradation of intracellular bacteria as part of a process referred to as xenophagy. Nod2 acts a sensor for invasive intracellular bacteria. As such Nod2 proteins have been show to accumulate adjacent to the plasma membrane after cells are co-incubated with *S. flexneri* (86). During *S. flexneri* infection, Nod2 appeared to recruit Atg16L1 to the site of bacterial entry, adjacent the plasma membrane. Mice expressing a frameshift mutation in *Nod2* comparable to that of the 3020insC NOD2 SNP, exhibited marked reduction in bacterial autophagy. Lymphoblasts homozygous for the CD-associated ATG16L1 T300A allele obtained from patients with CD, displayed a marked impairment in autophagosome formation in response to the Nod2 agonist muramyl dipeptide (86). It is unclear however, whether NOD2 mutation was present in the same cells.

These findings suggest that the observed impairment in autophagy in the intestine of patients with CD may occur as a result of a combined defect in NOD2 and ATG16L1. It is noteworthy that the interaction between Nod2 and Atg16L1 appeared to be unaffected by the *ATG16L1* T300A substitution associated with CD. However, this CD-associated Atg16L1 T300A variant was no different substitution. However, these cells were not genotyped for NOD2 mutations. These findings link *NOD2* and *ATG16L1*, both genes identified as risk factors for CD, in a shared pathway with implications for the pathogenesis of CD.

The combined effect of these mutations may have profound implications for innate immunity. The role of xenophagy in the epithelium may be twofold. Firstly, degradation of intracellular bacteria such as *S. flexneri*, *Salmonella* spp., and *L. monocytogenes* may reduce spread of these pathogens to uninfected cells. However, the significance of autophagic clearance of intracellular bacteria in limiting infections is unclear. Secondly, autophagy is a key element in the processing of bacterial antigen for presentation to T lymphocytes (87). It therefore seems plausible that combined *NOD2* and *ATG16L1* mutations may result in impaired adaptive immune responses to potentially pathogenic bacteria in the gut. Conversely, the effect could conceivably result in inappropriate immune responses directed against bacterial commensals that are normally tolerated in most people. The role for a defect in innate immunity due to defective autophagy is strengthened by the association of immunity-related GTPase family M (*IRGM*) with CD.

### IRGM

The small GTPase encoded by the human *IRGM* gene is thought to direct autophagy in the degradation of intracellular pathogens. So far, the only cellular proteins, which include Atg5 and Atg10, that have been shown to interact with Irgm, are all involved in the initial stages of autophagosome assembly (88). *IRGM* may limit viral infection directly, through autophagic clearance, and indeed a plethora of viral proteins target *IRGM* to disrupt autophagy and promote viral replication (88). Perhaps more important is the role of Irgm-mediated xenophagy in antigen processing and presentation to generate effective adaptive immune responses against bacteria invading the cell. Aside from antiviral immunity, *IRGM* is a significantly limiting intracellular bacterial infections has been well demonstrated. In mice, *Irgm1* mediates IFN-γ-induced autophagy required for the degradation of intracellular mycobacteria in macrophages (89). Similarly, human THP-1 macrophages overexpressing *IRGM* displayed decreased numbers of intracellular adherent-invasive *E. coli* (AIEC) (90).

Early studies initially associated genetic variation in the *IRGM* region with a susceptibility to CD (68). Further analysis revealed three *IRGM* coding sequence variants, two of which could not be associated with CD (91). The third *IRGM* risk variant identified was a synonymous exonic SNP which does not result in alteration to amino acid sequence or splicing sites (92). It is a deletion polymorphism located upstream of the *IRGM* coding region. The effect of this deletion polymorphism was cell type-dependent, and in a colonic cell line actually increased *IRGM* expression compared to the reference sequence used (92). Conversely, the aforementioned synonymous *IRGM* variant does in fact contain an altered miRNA binding site (93). As a result, binding of regulatory miR-196 is significantly impaired. To link a novel mechanism by which *IRGM* expression is potentially dysregulated in CD, Brest et al. analyzed the expression and localization of IRGM and miR-196 in the colonic mucosa (93). Expression of *IRGM* within the colonic epithelium is high under normal conditions, but is reduced during inflammation while epithelial miR-196 expression increases. In those patients with CD possessing the *IRGM* risk allele, no similar reduction of *IRGM* occurred during active inflammation, presumably due to inability of miR-196 to bind *IRGM* transcript. While overexpression of *IRGM* results in increased autophagic activity during AIEC infection in human macrophages, epithelial cells appear to respond differently (90). Increased autophagy in *IRGM* overexpressing HEK293 cells appeared to encourage proliferation of AIEC within autophagic vacuoles (93).

The functional consequence of *IRGM* overexpression in CD remains unclear. Invasive species of *E. coli*, including AIEC, have been observed to colonize the ileal mucosa of patients with CD in greater numbers than normal controls (94, 95). Inefficiency in the clearance of invasive pathogens via autophagy in the intestinal mucosa of patients with CD may result in persistent infection. This is supported by *in vitro* evidence suggesting that impaired autophagy due to ATG16L1, NOD2 and IRGM mutation in macrophages results in increased TNF-α and IL-6 secretion (90). This could conceivably lead to exacerbation of the initial inflammation and tissue injury allowing greater bacterial penetrance into the mucosa in IBD.

### PTPN2

Another gene recently identified as a risk factor in the development of IBD is T cell protein tyrosine phosphate non-receptor type 2 (*PTPN2*). An SNP (rs2542151) upstream of the coding region of PTPN2 has been associated with susceptibility to both CD (70, 96) and UC (72). A second SNP (rs1893217) located within intron 7 of the *PTPN2* gene has also been associated with CD (97, 98). The protein encoded by *PTPN2* appears to be an important negative regulator of signaling induced by pro-inflammatory cytokines such as IFN-γ and IL-6 through its ability to dephosphorylate STAT1 and STAT3 *in vitro* (99). *Ptpn2* knock-out mice display severe defects in T cell development and from an early age develop a lethal systemic inflammation, with high serum IFN-γ and TNF-α (100–102). It is interesting to note that *Ptpn2*^+^ mice display greater susceptibility to DSS than wild-type *Ptpn2* mice, suggesting that *Ptpn2* negatively regulates inflammation (103). Expression of *PTPN2* in human T84 colonocytes is enhanced by IFN-γ and TNF-α and accordingly *PTPN2* mRNA levels are increased in the inflamed mucosa of patients with CD (104, 105). Since the link between defective autophagy and CD was identified, a potential role for *PTPN2* in the regulation of autophagy has also been investigated. Autophagy in wild-type T84 colonic epithelial cells was induced by 24 h challenge with IFN-γ and TNF-α, indicated by increased LC3B-II protein and LC3B-labeled puncta (106). Knock-down of *PTPN2* with specific siRNA in the T84 cell line increased both mTOR phosphorylation and beclin 1 expression but simultaneously abrogated IFN-γ/TNF-α-induced up-regulation of Atg5 and Atg7. Consequently, fewer larger LC3B-labeled autophagosomes were observed following *PTPN2* siRNA knock-down, suggesting autophagosome formation was defective in *PTPN2* deficient cells. To assess the contribution of the rs2542151 *PTPN2* variant to the autophagy impairment observed in CD, Scharl et al. cultured lamina propria fibroblasts from the colon of patients with CD. Stimulation of fibroblasts with both IFN-γ and TNF-α increased autophagosome formation in wild-type *PTPN2* cells but this effect was absent in fibroblasts carrying the *PTPN2* CD-associated variant. The mechanisms by which *PTPN2* influences autophagy remain unclear. It has been suggested that PTPN2 exerts its autophagy-promoting effect by repressing mTOR activation via the EGFR-PI(3)K-AKT pathway following its activation by IFN-γ and TNF-α (107).

The role of *PTPN2* in coupling autophagy to pro-inflammatory signaling may mean that *PTPN2* is crucial in maintaining the integrity of the intestinal barrier against pathogens. Scharl et al., showed that *PTPN2* mutation or deficiency reduced the localization of *L. monocytogenes* to autophagosomes in colonic epithelial cells or fibroblasts (107). Whether the inclusion and/or clearance of *L. monocytogenes*, or other invasive bacteria such as AIEC, in autophagosomes in *PTPN2* deficient cells is affected by IFN-γ or TNF-α is yet to be determined. It is plausible therefore, that *PTPN2* risk allele associated with CD, in combination with *ATG16L1* and *NOD2* mutants, permits greater persistence and spread of intracellular pathogens, possibly contributing to a an increased susceptibility to chronic inflammation (76, 82). Aside from the role of *PTPN2* in handling intracellular bacteria, *PTPN2* may also regulate the permeability of the intestinal barrier. Deficiency of PTPN2 in T84 colonocytes increased the permeability of *in vitro* epithelial monolayers to ionic and macromolecular flux. Increased permeability was associated with a reduction in the expression of occludin and zona-occludens-1, proteins typically associated with epithelial tight junctions and key regulators of paracellular permeability. Recent evidence indicates a role for autophagy in the regulation of cell–cell adhesion in human intestinal epithelial cells stimulated with TNF-α. Pharmacological inhibition of TNF-α-induced autophagy resulted in a reduction in the protein levels of functional integrin β1 subunit (108). It is possible therefore that autophagy is responsible for the removal and recycling of cellular adhesion and tight junction proteins to facilitate regeneration of the intestinal epithelium after acute inflammation. Determining the full extent to which the CD-associated *PTPN2* mutation and the resultant autophagic defect contributes to chronic inflammation promises to yield valuable insight into the cellular physiology of the intestine in both health and chronic disease.

### XBP1

XBP1 is induced by ER stress and forms part of the unfolded protein response. One of the mechanisms used to regulate ER stress induced by misfolded protein in the ER is autophagy (9). The identification of two *XBP1* SNPs associated with CD and UC suggests a link between IBD and the ER stress response (109). The two *XBP1* variants were associated with hypomorphic XBP1 expression indicating that the XBP1-mediated ER stress response by is defective in patients with IBD.

Knock-out mice generated with an *XBP1* deletion restricted to the epithelial cells of the large and small intestine spontaneously develop mild small intestinal inflammation. *XBP1*^−/−^ Paneth cells and to a lesser extent, goblet cells, undergo ER stress and consequently undergo apoptosis. ER stress induced by *XBP1* deletion resulted in the depletion of Paneth cells in the small intestine but not in the colon. However, *XBP1*^−/−^ mice were vulnerable to the induction of DSS-colitis. *XBP1*^−/−^ mice displayed wasting, rectal bleeding, mucosal erosion, and leukocyte inflammation to a greater extent than did wild-type mice following 5 days of 4.5% DSS-administration (109). The consequence of hypomorphic *XBP1* mutation in human IBD patients may therefore result in impaired anti-microbial activity of Paneth cells. When combined with the aforementioned *ATG16L1* mutation in CD, defective *XBP1* may further promote deleterious interactions with the intestinal microbiota.

### ULK1

Following the identification of autophagy-related genes such as *ATG16L1*, *NOD2*, and *IRGM* a GWAS study was conducted to identify additional risk factors among known autophagy genes. An SNP in one these candidate genes, *ULK1*, was significantly more frequent among patients with CD (110). Mammalian *ULK1* is homologous to the yeast *Atg1* and its expression is ubiquitous in most tissues. Together with Atg13, and FIP200, ULK1 is an essential member of a large protein complex whose activation initiates the formation of the autophagosome. Autophagy induction under conditions of high glucose abundance is repressed by interaction between the autophagy regulator mTORC1 and ULK1, preventing phosphorylation of the latter. During glucose starvation, ULK1 associates instead with AMPK, resulting in phosphorylation of ULK1 and subsequent activation of autophagy (19, 111).

To best assess the biological function of *ULK1 in vivo*, a knock-out mouse model was developed. Interestingly, *ULK1*^−/−^ mice exhibited normal autophagy in response to nutrient deprivation suggesting that autophagy induction may still occur through Ulk2 signaling (112). The effect of *ULK1* deletion in autophagy-induced by other mechanisms however is yet to be determined. Invasion of intestinal epithelial cells by bacterial pathogens such as AIEC may be facilitated by impairment ULK, thereby promoting persistent intestinal inflammation.

### LRRK2

A polymorphism in the LRR kinase 2 gene (*LRRK2*) has been associated with susceptibility to CD (70, 71). However, knowledge of its contribution to the pathogenesis of IBD is limited. The expression of *LRRK2* is predominantly limited to monocytes, dendritic cells, and lymphocytes (113). Furthermore *LRRK2* expression can be up regulated in human PBMCs through IFN-γ stimulation. Since IFN-γ dominates the mucosal cytokine profile of patients with CD (114), it follows that *LRRK2* expression is higher in the inflamed mucosa of patients with CD relative to non-inflamed regions (113). Although mutations in *LRRK2* are known to confer risk of developing CD and also Parkinson’s disease, little evidence for its pathological function exists. However, *LRRK2* has relatively recently been implicated in the regulation of autophagic pathways. *LRRK2* co-localized with autophagolysosomes and multivesicular bodies, both structures formed by autophagy (115). In contrast to the other autophagy-related genes associated with CD, *LRRK2* appears to function as a negative regulator of autophagy. Cells transfected with siRNA specific for *LRRK2* exhibited enhanced basal autophagic activity, and enhanced starvation-induced autophagy (115). In neuronal cells, poorly controlled autophagy can result in autophagic stress. Neuronal cells transfected with Parkinson’s disease-associated *LRRK2* variants exhibited increased autophagic vacuole formation and accumulation of protein aggregates (116). However, the mutations associated with both Parkinson’s and CD is quite different. Mutation in the *LRRK2* kinase domain is associated with Parkinson’s disease whereas the CD-associated *LRRK2* variant results in less functional protein (117). To assess the functional consequences of the CD-associated mutant an experimental animal model was utilized. C57BL/6 mice generated with *Lrrk2* deficiency did not develop spontaneous intestinal pathology (117). However, following 8 days of DSS-administration, *Lrrk2*^−/−^ mice exhibited more severe colitis than mice with functional Lrrk. Notably, the pathology induced by DSS in *Lrrk2*^−/−^ mice was penetrated into the muscle layer in a similar fashion to CD lesions. Liu et al. identified a potential role for *LRRK2* in regulating pro-inflammatory cytokine secretion that likely contributes to the pathology of CD. However, the effect of reduced LRRK2 on autophagy, and the mechanism through which it occurs, remains to be investigated in this model.

The bulk of our understanding of autophagy mechanisms is derived from experimental work performed using starvation-induced autophagy as a model. It is assumed that the general mechanisms of autophagy will be conserved for the most part among autophagic processes induced by other stimuli. Naturally, alternative autophagy pathways have been observed. For example, the induction of autophagy by invasive bacteria may occur through the mTORC1 pathway, or independently of this pathway depending on bacterial species (17). It is noteworthy that starvation-induced autophagy also contributes to host defense in the event of infection with *Shigella* or *Salmonella* species (118). The mechanisms for starvation-induced autophagy, which may be somewhat non-specific and those regulating degradation of specific proteins and intracellular structures may also differ. Given the involvement of autophagy in a broad spectrum of cellular processes, it is likely that a wide range of receptors specific for various proteins and organelles exists. Several receptors mediating the targeting of protein aggregates to the internal membrane of the autophagosome have already been identified, perhaps the most prominent being p62, and more receptors are likely to be identified in the future (119).

## Conclusions and Future Directions

In this review, we have discussed in detail, the most important gene pathways that have been shown to be important in mediating autophagy in intestinal homeostasis. Research work in the area of autophagy suggests that autophagy is not only an important player in the pathogenesis of IBD but also a pathway that merges intestinal innate immune sensing, microbial immune response, and ER stress response. In all the above mentioned cellular responses the intestinal epithelium, including the goblet cells and Paneth cells, seems to orchestrate major signaling events. While current studies are not conclusive about the inter-mingled pathways connecting innate immunity, autophagy, and ER stress, future studies will unravel the common pathways. While the overall gene mutations/polymorphisms associated with autophagy and their importance in predisposition to sub types of IBD are understood, the exact triggers for defective autophagy are not understood yet. For example, mice hypomorphic for Atg16L1 need a viral infection trigger for inducing colitis-like pathology. Future research work will focus on identifying specific environmental triggers that elicit defective autophagy responses in disease.

While much of our understanding of autophagy mechanisms comes from *in vitro* systems using either yeast or mammalian cell lines, the significance of autophagy in pathology has mostly been derived from experimental animal models. Using animals to model defective autophagy mechanisms may however, introduce an additional layer of complexity in our attempts to understand cell type-specific defects in autophagy. Autophagy appears to be ubiquitous through virtually all cell types and tissues and is employed in a wide range of functions. The mammalian intestinal mucosa includes several types of structural cells in addition to a large concentration of resident immune cells. Functions such as maintaining cytoplasmic stores of amino acids and removal of defective mitochondria are likely to be employed by most cell types. However autophagy is also involved in more specialized processes which are restricted to certain types of other cells. For example, autophagic mechanisms contribute to the secretion of anti-microbial peptides and cytokines. Mutation of a single autophagy gene may therefore be predicted to result in a broad spectrum of pathological phenotypes across the various cells of a single tissue. Considering the ubiquity of autophagy throughout human tissues future attempts to modulate pathologic immune responses caused in part by dysfunctional autophagy, may be difficult. Investigating the contribution of dysfunctional autophagy in each cell type of the intestinal mucosa may therefore be necessary. Nevertheless, autophagy and its key role in regulating homeostasis in the intestinal mucosa make it an attractive target for exploring future avenues for therapy. Given the rapidly growing interest in the role autophagy in human disease it seems likely that the next few years will bring about a wealth of exciting new research findings in IBD and autoimmune disease in general.

## Conflict of Interest Statement

The authors declare that the research was conducted in the absence of any commercial or financial relationships that could be construed as a potential conflict of interest.
